# An Analysis of the Effect of Stroke on Health-Related Quality of Life of Older Adults With Coronary Heart Disease Who Take Aspirin

**DOI:** 10.7759/cureus.43611

**Published:** 2023-08-16

**Authors:** Adenike R Sulaiman, Helen Oletu, Assumpta Chike, Chinenye Ani, Francis Twumasi, Ugochinyere Ikechukwu, Okelue E Okobi, Abubakar M Sani, Faith C Onyeaka, Abigail O Dan-Eleberi, Joy Iroro

**Affiliations:** 1 Internal Medicine, Lagos State University College of Medicine, Lagos, NGA; 2 Medicine and Surgery, University of Benin, Benin City, NGA; 3 Public Health, University of Wolverhampton, Wolverhampton, GBR; 4 Internal Medicine, University of Science, Arts and Technology, Olveston, MSR; 5 Internal Medicine, Savanna La Mar Public General Hospital, Savanna La Mar, JAM; 6 Neurology, Hainan Medical University, Haikou, CHN; 7 Family Medicine, Alberta International Medical Graduates Association, Calgary, CAN; 8 Family Medicine, Larkin Community Hospital Palm Springs Campus, Miami, USA; 9 Family Medicine, Medficient Health Systems, Laurel, USA; 10 Family Medicine, Lakeside Medical Center, Belle Glade, USA; 11 Internal Medicine, Kaduna State Ministry of Health, Kaduna, NGA; 12 Medical Laboratory Science, Madonna University, Elele, NGA; 13 Internal Medicine, University of Ghana Medical School, Accra, GHA; 14 Internal Medicine, All Saints University School of Medicine, Roseau, DMA

**Keywords:** mental health, physical health, respondents, hrqol, health-related quality of life, quality of life, aspirin, chd, coronary heart disease, stroke

## Abstract

Objective: The aim of this study was to examine the impact of coronary heart disease (CHD) on health-related quality of life (HRQoL) among individuals taking aspirin, as well as to explore the potential association between stroke and CHD on HRQoL.

Method: A total of 17,106 respondents aged 50 years and above who reported using aspirin on "some days" or "daily" were included in the analysis. Among them, 4,036 individuals had a history of coronary heart disease. We utilized the Chi-square test to assess the proportion of individuals with CHD who reported poor self-rated health and experienced poor HRQoL in four domains: physical health, mental health, physical and mental health combined, and the number of days limited by poor health. Logistic regression was employed to investigate the interaction between stroke and CHD concerning the quality of life.

Result: Among adults aged 50 years and above using aspirin, those with CHD tended to be older (68.7 years ± 0.37 vs 66.6 ± 0.24), had a higher proportion of male respondents (60.0% vs 45.1%), and were mostly of white ethnicity (77.4% vs 76.2%). The group with CHD reported significantly poorer self-rated health compared to those without CHD (52.1% vs 25.6%, p<0.001), along with a higher prevalence of poor physical health (55.3% vs 42.7%, p<0.001) and poor mental health (50.2% vs 40.4%, p = 0.033) in comparison to aspirin users without CHD. However, there was no statistically significant association between stroke and CHD concerning the impact on all domains of quality of life (p>0.05).

Conclusion: Our findings indicate that individuals aged 50 years and above with CHD who are using aspirin experience a lower quality of life in both the physical and mental health domains when compared to their counterparts without CHD. Furthermore, there was no significant interaction between stroke and CHD in relation to the impact on HRQoL in this study.

## Introduction

The CDC defines health-related quality of life (HRQoL) as an individual’s perceived physical and mental health, both of which are affected by functional status, health conditions, social support, and community-level policies [1]. Coronary heart disease (CHD) and stroke, through functional limitations, are health conditions that reportedly impact the quality of life of older adults [2-4]. Among patients with CHD, studies have shown that sleep disturbance, social support, and other characteristics of the disease have an impact on the quality of life of these individuals [5,6]. Demographic factors such as income and level of education have also been implicated as negative correlates of HRQoL in patients with CHD [6]. Similarly, the sequelae of stroke, such as cognitive impairment, aphasia, and vision loss [4,7], as well as sociodemographic factors such as age and sex, have been shown to correlate with HRQoL among stroke populations [8].

Aspirin, a medication used for the prevention of cardiovascular disease in patients with stroke and coronary heart disease, has also been shown to affect not only the quantity of life through impacting life expectancy and disease incidence but also the quality of life. Djatche et al. reported that aspirin use may improve the quality-adjusted life year of patients following a primary cardiovascular event [9]. However, studies have shown that aspirin has mixed effects on the quality of life of populations as they grow older [10]. While different studies have evaluated how various aspects of CHD or stroke, sociodemographic factors, and mood disorders affect HRQoL among these populations, no previous studies have evaluated how stroke status interacts with CHD status for patients on aspirin as it pertains to HRQoL. This is important given the current interventions in place to address HRQoL among populations with CHD.

One such intervention is a cardiac rehabilitation program. This program has been shown to be beneficial to patients with CHD [11,12]. It involves education and physical exercise among other measures. A similar but slightly different intervention has been successfully implemented in stroke populations through self-management [13] and aerobics programs [14], but some of these programs have been unsuccessful in improving HRQoL [15]. It is therefore reasonable to hypothesize that in patients with CHD on aspirin for secondary prevention, stroke may have an interaction effect on their quality of life and interventions for these select groups of people may not have the same quality of life effects as expected. Therefore, this paper seeks to assess the HRQoL of patients 50 years and above with CHD on aspirin using a nationally representative sample and to analyze the effect of having CHD and being on aspirin on HRQoL if they do not have stroke.

## Materials and methods

Study design

The behavioral risk factor surveillance system (BRFSS) is population-based surveillance of non-institutionalized US adults aged ≥18 years with various health conditions, including CHD and stroke. The survey collects data on sociodemographic status, health-related quality of life, including physical and mental health, and medication use, such as aspirin. The data were collected monthly by trained interviewers using an independent probability sample of households. A detailed description of the survey design and random sampling procedures is available elsewhere [16].

Study population

Among 274,238 adults aged 50 years or older, 1.3% (n = 3,539) did not respond to the survey question on CHD. Of the remaining 270,699 adults, 17,106 respondents representing about seven million older adults ≥50 years who reported using aspirin on “some days” or “daily”. Among them, 4,036 reported having a history of coronary heart disease.

Measures

Sociodemographic and Cardiovascular Risk Factors

Sociodemographic variables considered in this analysis include age, sex, race, level of education, employment status, and body mass index (BMI). They were reported as follows: age as mean (standard error); sex was categorized as male and female; level of education was categorized into less than college, college degree, and above college degree; race was categorized as white, black, and others; employment status was categorized as employed or unemployed; and BMI was categorized as <25, ≥25 but <30 and >30 kg/m^2^.

Cardiovascular risk factors were smoking status, diabetes mellitus, and hypertension. Diabetes mellitus and hypertension were categorized as “yes” if participants stated they had the condition and “no” if they did not; and smoking status was categorized as current, never, or former smokers.

Stroke status

Respondents with stroke were coded as “yes” if they answered yes to ever being diagnosed of stroke and “no” if not ever being diagnosed of stroke.

Health-related quality of life

Respondents were asked questions related to their overall health status and HRQoL. Concerning their overall health, they were asked, “Would you say that in general your health is excellent, very good, good, fair, or poor?” and it was coded as good health if they responded as either excellent, very good, or good, and poor health if they responded as fair or poor. Respondents were asked three questions concerning their HRQOL. (1) “Now thinking about your mental health, which includes stress, depression, and problems with emotions, for how many days during the past 30 days was your mental health not good?”. (2) “Now thinking about your physical health, which includes physical illness and injury, for how many days during the past 30 days was your physical health not good?”. (3) “During the past 30 days, for about how many days did poor physical or mental health keep your from doing your usual activities, such as self-care, work, or recreation?”. From these, we created four dichotomous quality-of-life domains that categorized persons into two mutually exclusive groups depending on whether they did or did not report ≥ 14 unhealthy days. These variables were mental and physical. Physical activities were limited by poor HRQoL and overall health. These four variables and their construct validity have been described elsewhere [17]. The demarcation point of 14 unhealthy days defines a meaningful cut point for those reporting impaired HRQoL and corresponds to the upper 10-15% of the distribution for each of the healthy day's measures [18].

Statistical analysis

The statistical analysis was conducted in three steps. First, we applied sampling survey weight due to the complex survey methodology, and then we characterized the study population using descriptive analysis with weighted percentages reported. Next, we used Pearson Chi-square analysis to identify group differences in HRQoL by CHD on aspirin status and reported weighted percentages. Lastly, we evaluated the interaction of stroke on the HRQOL of respondents with CHD who are on aspirin using logistic regression, adjusting for sociodemographic and cardiovascular risk factors, with an adjusted odds ratio reported. Analysis was conducted with STATA 14.0 (StataCorp, College Station, TX) with statistical significance set at p<0.05 using a two-tailed test.

Ethical approval

BRFSS provides valuable insights that inform decision-making and deepen our understanding of health trends and disparities among the civilian, non-institutionalized population in the United States. The survey's data and information are available to the public through the website [16], falling under the public domain. As a result, this data can be utilized, copied, distributed, or published without requiring additional explicit permission or Institutional Review Board (IRB) review, as it involves the analysis of de-identified data and does not constitute Human Subjects Research (HSR) as defined at 45 CFR 46.102.

## Results

Table 1 shows the sociodemographic characteristics of the study population of adults aged 50 years and above. Among them, 4,036/17,106 (22.8%) reported having CHD and were on aspirin, with a mean (SE) age of 68.7 (0.37) years. As compared to those on aspirin who did not have CHD, there were older (68.7 years ± 0.37 vs 66.6 ± 0.24), more male respondents (60.0% vs 45.1%) and mostly white (77.4% vs 76.2%). In addition, CHD patients on aspirin as compared to their counterparts had less than a college education (50.1% vs 44.0%) and were less employed (81.6% vs 66.7%). However, they reported more stroke (19.6% vs 8.1%) as compared to those on aspirin who do not have CHD. The full details are shown below.

**Table 1:** Baseline characteristics of the study population. CHD: coronary heart disease, SE: standard error.

Variable	Respondents ≥50 years without CHD and were on aspirin n (%) = 13,070 (76.4%)	Respondents ≥50 years with CHD and were on aspirin n (%) = 4,036 (22.8%)
Mean age, year (SE)	66.6 (0.24)	68.7 (0.37)
Men	45.1%	60.0%
Race		
White	76.2%	77.4%
Black	14.0%	12.5%
Others	9.8%	10.1%
Level of education		
Less than college	44.0%	50.1%
College graduate	31.3%	31.6%
Above college	24.7%	18.3%
Employment status		
Unemployed	66.7%	81.6%
Employed	33.4%	18.4%
Body mass index (kg/m^2^)		
<25.0	24.5%	23.4%
25.0–29.9	39.2%	37.2%
≥30.0	36.3%	39.4%
Stroke status		
No stroke	91.9%	80.5%
Stroke	8.1%	19.6%
Diabetes mellitus		
Non-diabetic	72.8%	60.5%
Diabetic	27.2%	39.5%
Hypertension		
Non-hypertensive	37.5%	21.5%
Hypertensive	62.5%	78.5%
Smoking status		
Never	52.8%	36.8%
Current	11.5%	16.6%
Former	35.7%	46.5%

Table 2 shows the proportion of respondents ≥50 years of age on aspirin by CHD status based on reported HRQoL. In general, a higher proportion of adults ≥50 years of age with CHD who were on aspirin reported poor self-rated health as compared to their counterparts (52.1% vs 25.6%, p<0.001). With regards to specific HRQoL domains, a higher proportion of those who had CHD and were taking aspirin reported having poor physical health (55.3% vs 42.7%, p<0.001), poor mental health (50.2% vs 40.4%, p = 0.033), and ≥14 days of activity limitation by the poor quality of life (57.7% vs 47.3%, p = 0.025) as compared to their counterparts.

**Table 2:** Proportion of respondents by health-related quality of life measures. CHD: coronary heart disease.

Variable	Respondents without CHD who were on aspirin	Respondents with CHD who were on aspirin	p-value
Poor self-rated health	25.6%	52.1%	Less than 0.001
Poor physical health % ≥14 days	42.7%	55.3%	Less than 0.001
Poor mental health % ≥14 days	40.4%	50.2%	0.033
Activity limited days % ≥14 days	47.3%	57.7%	0.025
Overall unhealthy days % ≥14 days	47.8%	54.5%	0.288

**Figure 1:**
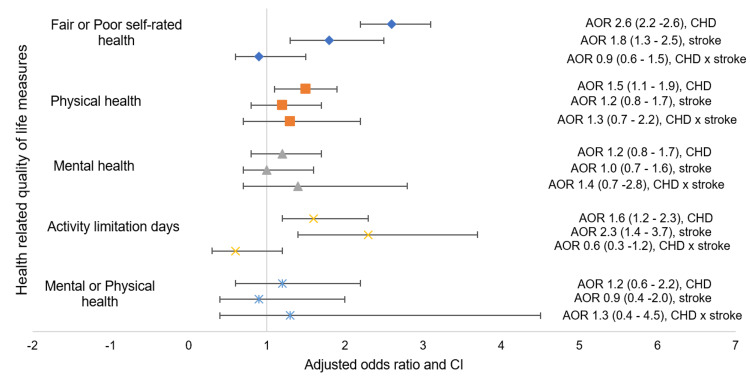
Forest plot of the interaction analysis of stroke and aspirin on quality of life. *Adjusted for sex, race, education, employment status, BMI, diabetes mellitus, hypertension, and smoking status. michd: myocardial infarction/coronary heart disease, michd +: myocardial infarction/coronary heart disease alone, stroke +: stroke alone, michd + stroke +: interaction term of myocardial infarction/coronary heart disease and stroke, CHD: coronary heart disease.

In Figure 1, the main effects of having CHD and being on aspirin, as well as the interaction effect of stroke on HRQoL, are shown with covariates adjusted for. It shows that there is no statistically significant interaction between stroke on the relationship between having CHD and being on aspirin as it pertains to all domains of quality of life (p>0.05). The main effect of having CHD and being on aspirin on fair or poor self-rated effect (AOR: 2.6, 95% CI: 2.2-2.6), physical health (AOR: 1.5, 95% CI: 1.1-1.9), and >14% activity limitation days (AOR: 1.6, 95% CI: 1.2-2.3) was statistically significant. The main effect on mental health was, however, not statistically significant, and in addition, a similar non-significant relationship was seen when mental and physical health were evaluated in combination.

## Discussion

The objective of this study was to assess the HRQoL of adults ≥50 years with CHD who were on aspirin in comparison to those without CHD but on aspirin and subsequently evaluate if, in the absence of stroke as comorbidity, the effect of CHD and concurrent aspirin use contributes to poor quality of life. We found out that overall, as compared to those who were on aspirin and did not have CHD, a higher proportion of respondents with CHD who were on aspirin reported worse quality of life measures in the physical and mental domains of HRQoL. In the absence of stroke, CHD and concurrent aspirin were negatively associated with poor physical but not mental HRQoL domains.

This high proportion of poor physical HRQoL among respondents with CHD who were on aspirin was consistent with reports from other studies that have evaluated the effect of heart disease on the physical aspect of HRQoL. Staniute et al. reported a poor HRQoL associated with greater fatigue and decreased exercise capacity in patients with coronary artery disease [19]. In addition, Reddy et al. reported poor physical quality of life in patients with heart disease, with poorer quality in those who are obese and with diabetes mellitus [20]. Furthermore, in the absence of a comorbidity like stroke, which has a negative effect on the physical aspect of one’s quality of life, having CHD and being on aspirin was still associated with poor physical quality of life. This means that aside from stroke, CHD, or aspirin, as well as other comorbidities such as obesity, contribute to poor physical HRQoL. In our study, the population with CHD who were on aspirin had a higher BMI as compared to their counterparts, further supporting the assumption that other comorbidities aside from stroke negatively impact HRQoL [21], specifically in populations with heart disease [22].

With regards to the mental health aspect of HRQoL, respondents who had CHD and were on aspirin reported a high proportion of poor mental health HRQoL as compared to their counterparts. However, in the absence of stroke, having CHD and being on aspirin was not associated with a poor quality of life with regard to mental health. This was inconsistent with findings reported in a study that reported stroke as a negative correlate of the mental aspect of HRQoL among adults with CHD. However, this inconsistency may be due to the nature of the tool used in assessing the mental aspect of HRQoL, as the authors made use of a standardized quality of life scale (EuroQoL-5D (EQ-5D)), unlike the open survey questions used in this study [23,24]. Despite these differences in HRQoL evaluation tools, the poor mental aspect of HRQoL of patients with CHD has been linked to poor perception of illness with regards to control of the symptoms of CHD, consistency of information on CHD status, and timeline of progression or management of the disease [25]. Furthermore, the degree of associated comorbidities has also been implicated in the observed poor mental HRQoL in patients with CHD [26-29], and as such, despite the consideration of stroke in this analysis, a cumulative effect of other comorbidities such as older age, kidney disease, diabetes mellitus, and hypertension may be contributory to the high proportion of poor mental HRQoL reported by respondents.

The implication of the findings from this study is that in the design of interventions for patients with CHD, specific measures to improve the physical and mental aspects of HRQoL should be considered. For instance, weight loss programs to address associated comorbidities may be a priority, as these individuals would be more active with a lower BMI, which may improve their physical HRQoL. Other measures such as consistent education on the nature and etiology of their disease, the timeline of interventions, clear expectations of the management of symptoms, improved social support, and mitigating stressful life events may improve the mental aspects of HRQoL in CHD patients [27-29].

This study has a few strengths and some limitations. Strengths of this paper include the novel analysis of the interaction effect of stroke on the HRQoL of adults 50 years and above with CHD who are on aspirin as compared to their counterparts. It used weighted data to represent a national sample and was powered enough to find statistical significance. The limitations of this study include the nature of the quality of life assessment. Despite the prior use of these variables in previous analyses that reported health-related quality of life measures, these HRQoL measures may not be as valid as standardized questionnaires, and as such, care must be taken when making inferences. Other comorbidities such as chronic obstructive pulmonary disease, obstructive sleep apnea, peripheral artery disease, and chronic pain, which have been reported to impact HRQoL, were not controlled for in the interaction model. A temporal relationship between CHD and HRQoL is difficult to assume from survey data, and there is a risk of recall bias as respondents are asked questions that depend on a good recall.

## Conclusions

In conclusion, people with CHD on aspirin seem to report worse physical and mental HRQoL as compared to their counterparts. In the absence of stroke, having CHD and being on aspirin was associated with adverse physical health outcomes, including poor health-related quality of life. It is possible that other comorbidities, such as obesity, may affect this relationship aside from stroke. These interactions and effects of HRQoL should be considered when designing interventions to help patients with CHD manage their disease. Studies using validated questionnaires to further study the effects of comorbidities such as stroke on the quality of life of individuals with CHD who are on aspirin are needed.
